# Corrigendum: Exact Distribution of Linkage Disequilibrium in the Presence of Mutation, Selection, or Minor Allele Frequency Filtering

**DOI:** 10.3389/fgene.2020.00732

**Published:** 2020-08-11

**Authors:** Jiayi Qu, Stephen D. Kachman, Dorian Garrick, Rohan L. Fernando, Hao Cheng

**Affiliations:** ^1^Department of Animal Science, University of California, Davis, Davis, CA, United States; ^2^Department of Statistics, University of Nebraska Lincoln, Lincoln, NE, United States; ^3^School of Agriculture, Massey University, Wellington, New Zealand; ^4^Department of Animal Science, Iowa State University, Ames, IA, United States

**Keywords:** linkage disequilibrium, effective population size, mutation rate, selection, minor allele frequency filtering

In the original article, there was a mistake in Figure 1 as published. A wrong graph was used in Figure 1A when c = 0.01. The corrected Figure 1 appears below.

**Figure 1:**
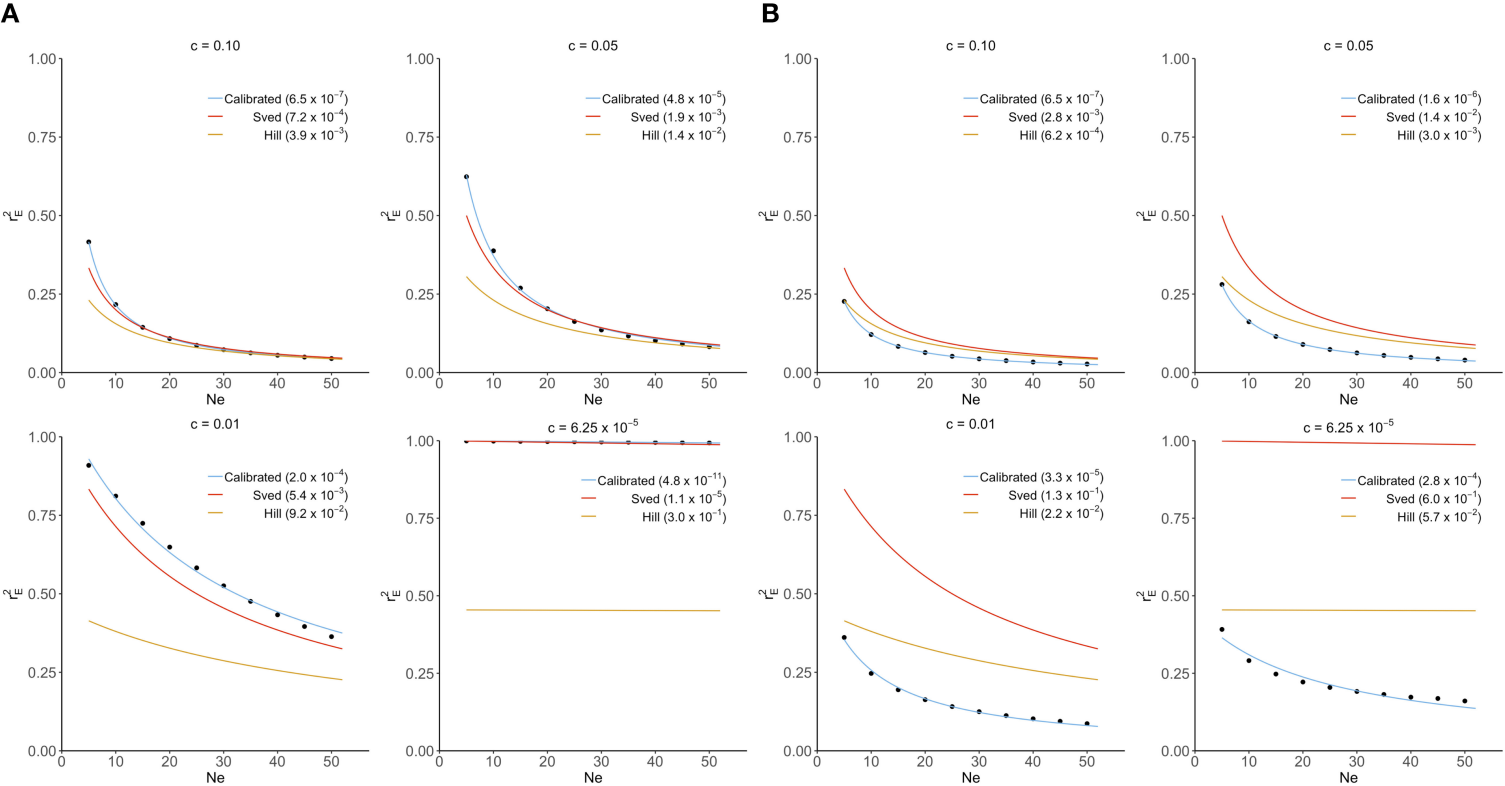
Comparison of Sved's and Hill's approximation to exact distribution of $r_{E}^{2}$ (scatter points) derived from transition-matrix approach. Mean square errors are shown in the parentheses. “Calibrated” denotes the non-linear regression formula derived from the transition-matrix approach. **(A)** In the absence of mutation and selection. **(B)** In the presence of mutation but no selection.

The authors apologize for this error and state that this does not change the scientific conclusions of the article in any way. The original article has been updated.

