# Supplementary material for: Exact Distribution of Linkage Disequilibrium in the Presence of Mutation, Selection, or Minor Allele Frequency Filtering
Source: Front Genet. 2020 Apr 21;11:362. doi: 10.3389/fgene.2020.00362 (PMC7212447; doi:10.3389/fgene.2020.00362)
Supplement: Supplementary file 1 [file Data_Sheet_1.PDF]

Supplementary Material

1 SUPPLEMENTARY TABLES AND FIGURES

1.1 Tables

Table S1. Comparisons of mean squared errors among the calibrated formula, Sved's formula and Hill's formula under different recombination rates (c) using validation data sets.

c	Calibrated	Sved	Hill
6.25×10^{-5}	7.39×10^{-4}	6.78×10^{-1}	8.31×10^{-2}
0.01	5.10×10^{-5}	7.01×10^{-2}	2.22×10^{-2}
0.02	1.31×10^{-5}	2.22×10^{-2}	9.46×10^{-3}
0.03	6.15×10^{-6}	1.01×10^{-2}	4.95×10^{-3}
0.04	3.86×10^{-6}	5.42×10^{-3}	2.91×10^{-3}
0.05	2.81×10^{-6}	3.24×10^{-3}	1.85×10^{-3}
0.06	2.21×10^{-6}	2.08×10^{-3}	1.24×10^{-3}
0.07	1.82×10^{-6}	1.41×10^{-3}	8.61×10^{-4}
0.08	1.53×10^{-6}	9.84×10^{-4}	6.17×10^{-4}
0.09	1.31×10^{-6}	7.09×10^{-4}	4.52×10^{-4}
0.1	1.13×10^{-6}	5.22×10^{-4}	3.37×10^{-4}
0.2	3.24×10^{-7}	4.11×10^{-5}	2.60×10^{-5}
0.3	1.13×10^{-7}	1.95×10^{-6}	6.04×10^{-7}
0.4	4.32×10^{-8}	9.39×10^{-7}	1.77×10^{-6}
0.5	1.68×10^{-8}	5.56×10^{-6}	6.73×10^{-6}

1.2 Figures

A_1B_1	A_1B_2	A_2B_1	A_2B_2
0	0	0	4
0	0	1	3
0	0	2	2
0	0	3	1
0	0	4	0
0	1	0	3
0	1	1	2
0	1	2	1
0	1	3	0
0	2	0	2
0	2	1	1
0	2	2	0
0	3	0	1
0	3	1	0
0	4	0	0
1	0	0	3
1	0	1	2
1	0	2	1
1	0	3	0
1	1	0	2
1	1	1	1
1	1	2	0
1	2	0	1
1	2	1	0
1	3	0	0
2	0	0	2
2	0	1	1
2	0	2	0
2	1	0	1
2	1	1	0
2	2	0	0
3	0	0	1
3	0	1	0
3	1	0	0
4	0	0	0

Figure S1. All possible combinations of haplotype frequency counts for a diploid population when $N_e = 2$.

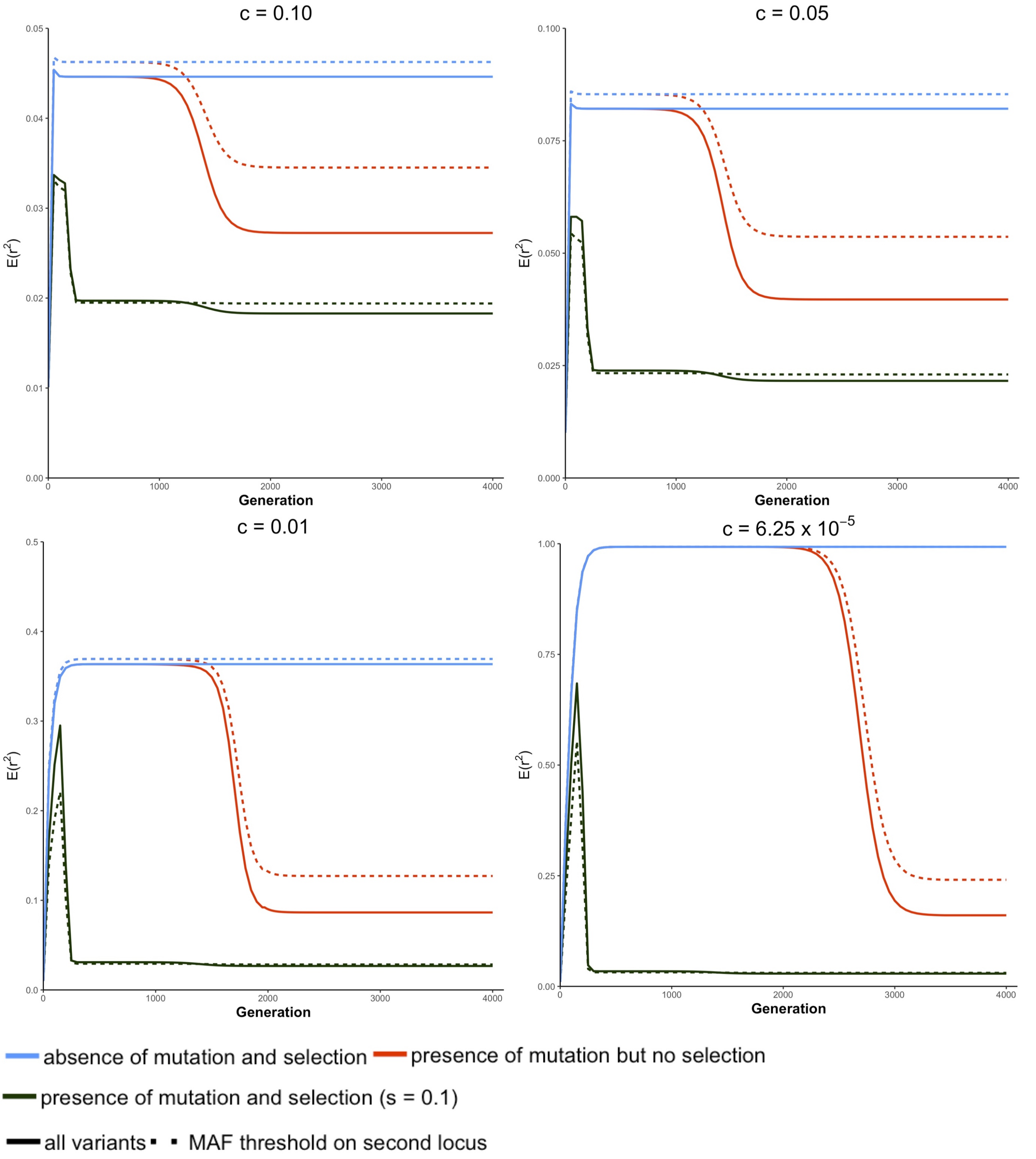

Figure S2. Expected value of LD $\left(E(r^2)\right)$ over generations in the absence or presence of filtering by MAF (i.e., MAF ≥ 0.05 for locus B) under three different conditions for a population of effective size $N_e = 50$.
